# An early prediction model for chronic kidney disease

**DOI:** 10.1038/s41598-022-06665-y

**Published:** 2022-02-17

**Authors:** Jing Zhao, Yuan Zhang, Jiali Qiu, Xiaodan Zhang, Fengjiang Wei, Jiayi Feng, Chen Chen, Kai Zhang, Shuzhi Feng, Wei-Dong Li

**Affiliations:** 1 Department of Genetics, College of Basic Medical Sciences, Tianjin Medical University, Tianjin, 300070 China; 2 School of Public Health, Tianjin Medical University, Tianjin, China; 3 Tianjin General Hospital, Tianjin Medical University, Tianjin, 300052 China

**Keywords:** Chronic kidney disease, Risk factors, Epidemiology, Outcomes research

## Abstract

Based on the high incidence of chronic kidney disease (CKD) in recent years, a better early prediction model for identifying high-risk individuals before end-stage renal failure (ESRD) occurs is needed. We conducted a nested case–control study in 348 subjects (116 cases and 232 controls) from the “Tianjin Medical University Chronic Diseases Cohort”. All subjects did not have CKD at baseline, and they were followed up for 5 years until August 2018. Using multivariate Cox regression analysis, we found five nongenetic risk factors associated with CKD risks. Logistic regression was performed to select single nucleotide polymorphisms (SNPs) from which we obtained from GWAS analysis of the UK Biobank and other databases. We used a logistic regression model and natural logarithm OR value weighting to establish CKD genetic/nongenetic risk prediction models. In addition, the final comprehensive prediction model is the arithmetic sum of the two optimal models. The AUC of the prediction model reached 0.894, while the sensitivity was 0.827, and the specificity was 0.801. We found that age, diabetes, and normal high values of urea nitrogen, TGF-β, and ADMA were independent risk factors for CKD. A comprehensive prediction model was also established, which may help identify individuals who are most likely to develop CKD early.

## Introduction

Chronic kidney disease (CKD), especially its complications, has posed a serious threat to public health worldwide. The global all-age mortality rate from CKD increased by 41.5% between 1990 and 2017^1^. A cross-sectional study showed that the prevalence of chronic kidney disease in China was approximately 10.8%^2^, which means that there were approximately 119.5 million CKD patients in China.

To date, certain risk factors are highly associated with chronic kidney disease, including age^3^, female sex^4^, obesity^5^, and diabetes mellitus^6^. Recently, several biomarkers associated with CKD were found. A few previous studies have shown that elevated ADMA (asymmetric dimethylarginine) levels could cause renal damage^7^. Several studies have pointed out that ADMA is a powerful biomarker for predicting CKD mortality^8–10^. It has also been shown that NFAL (neutrophil gelatinase-associated lipocalin) expression levels appear to correlate with the degree of renal dysfunction, which may help to identify patients at high risk for a more rapid decline in renal function^11^. Furthermore, the decrease in serum CysC (cystatin C) is correlated with the decrease in eGFR concentration^12^. It has been speculated that CysC could be used together with serum creatinine as a new biomarker or as a substitute for serum creatinine to better identify the occurrence of kidney disease in the general population^13,14^. TGF-β (transforming growth factor-β) is the main regulator of tubular interstitial fibrosis^15^, and TGF-β signaling can influence a few important renal injury responses in other growth factor signaling pathways^16,17^, ultimately affecting the onset of CKD^18^. Previous studies have reported that more than 50 single nucleotide polymorphisms (SNPs) are associated with renal function indexes or CKD worldwide^19^.

The treatment of chronic kidney disease and renal failure is costly and rarely effective. However, less than 5% of patients with early CKD report awareness of their disease^20^. Once CKD can be diagnosed, glomerular damage has reached over 50% and is usually irreversible. Effective prediction of chronic kidney disease can be immensely useful in this aspect. Therefore, several CKD prediction models for different populations were^21–24^ introduced. Recently, a study developed equations for predicting CKD based on 34 multinational cohorts^25^. Nevertheless, few models have considered both genetic and nongenetic risk factors. Although many prediction models reached high prediction power in a relatively large population, early prediction [at least when eGFR > 60 mL/(min·1.73 m^2^)] is essential for CKD treatment and prevention. In this study, we developed genetic, nongenetic (including biomarkers), and comprehensive risk score prediction models for CKD in a nested case–control study.

## Results

**Figure 1:**
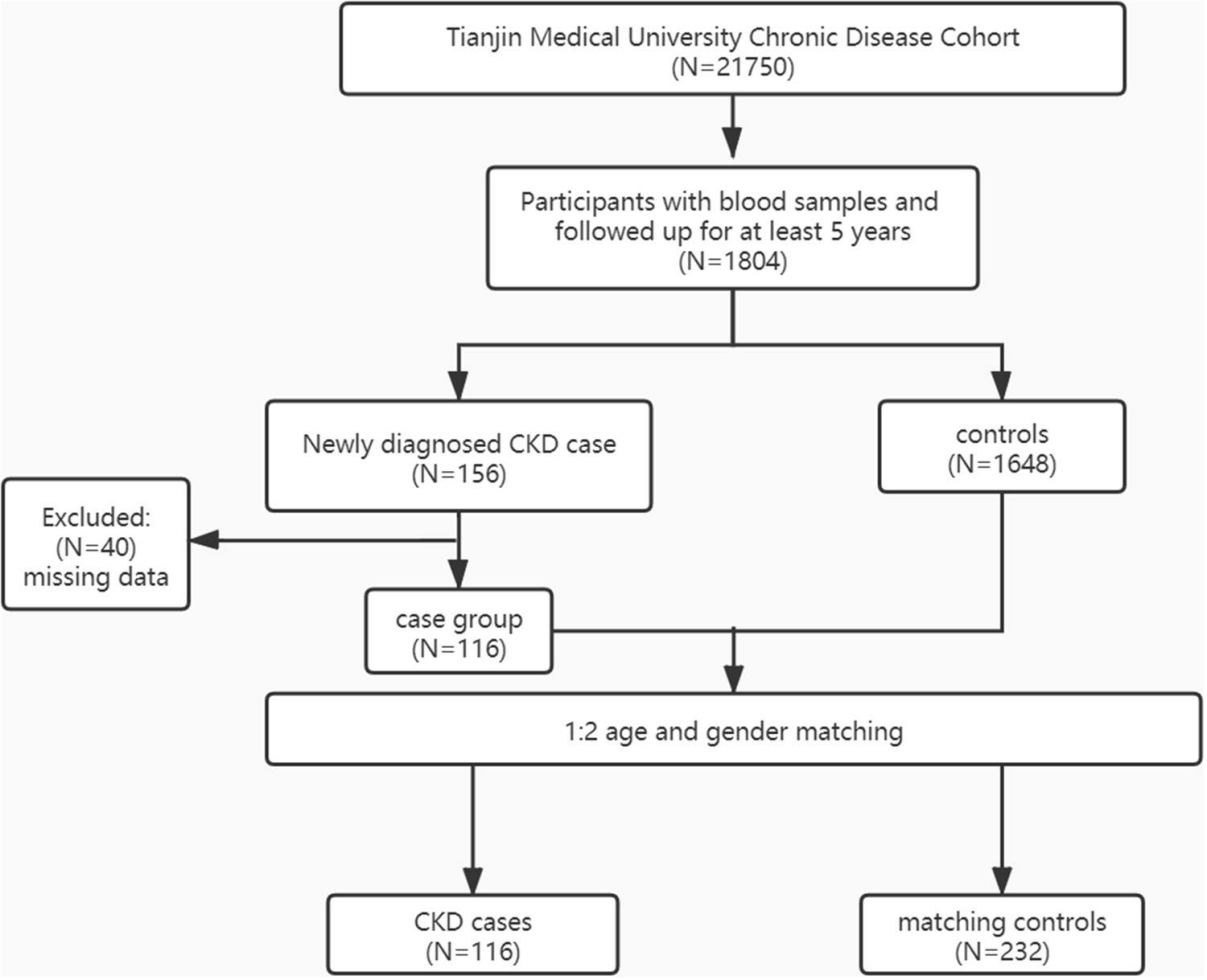
Flow chart of subjects in the nested case–control study.

In this nested case–control study, 348 participants (all had eGFR ≥ 60 mL/(min·1.73 m^2^) at baseline) were included (116 cases, 232 controls, subjects who reached eGFR < 60 mL/(min·1.73 m^2^) during the 5-year follow-up were considered “cases”) (Fig. 1) to build a 5-year risk prediction model for the onset of CKD. The baseline characteristics of the included participants in the nested case–control study are described in Table 1. The levels of fasting plasma glucose (FPG), total cholesterol (TC), urea nitrogen (BUN), serum creatinine (SCr), total protein (TP), globulin (GLB), systolic blood pressure (SBP), cystatin C (CysC), transforming growth factor-β (TGF-β), and asymmetric dimethylarginine (ADMA) in the CKD group were significantly higher than those in the controls. The age of the CKD group was significantly higher than that of the non-CKD group, and the incidences of type 2 diabetes and hyperuricemia were higher than those of the non-CKD group (Table 1). In addition, triglyceride (TG), serum uric acid (SUA) and body mass index (BMI) levels in the CKD group were higher than those in the non-CKD group, but the differences were not statistically significant.

**Table 1:** Baseline characteristics of subjects in the nested case–control study.

	Total (n = 348)	CKD group (n = 116)	Non-CKD group (n = 232)	*P* value
Men (%)	260 (74.7%)	84 (70%)	176 (77.2%)	< 0.001
Age (years)	63.27 ± 10.09	63.96 ± 7.74	63.33 ± 7.14	0.947
eGFR	82.85 ± 15.72	88.42 ± 14.74	72.40 ± 11.79	0.007
FPG (mmol/L)	5.11 ± 1.04	5.28 ± 1.13	5.02 ± 0.98	< 0.001
TC (mmol/L)	5.04 ± 0.88	5.22 ± 0.85	4.95 ± 0.89	0.006
TG (mmol/L)	1.75 ± 1.34	1.81 ± 1.65	1.72 ± 1.15	0.508
BUN (mmol/L)	5.45 ± 1.11	5.94 ± 1.11	5.20 ± 1.03	< 0.001
SCr (μmmol/L)	83.11 ± 14.32	89.82 ± 14.39	79.58 ± 12.99	0.009
SUA (μmmol/L)	335.6 ± 76.33	344.9 ± 81.27	330.8 ± 73.40	0.100
TP (g/L)	75.41 ± 4.30	75.94 ± 5.17	75.13 ± 3.74	0.005
ALB (g/L)	45.74 ± 2.62	45.57 ± 2.69	45.84 ± 2.59	0.938
GLB (g/L)	29.68 ± 3.41	30.38 ± 3.97	29.32 ± 3.01	0.003
ALT (IU/L)	25.93 ± 3.30	24.44 ± 10.39	26.71 ± 8.60	0.030
TBIL (μmol/L)	14.38 ± 4.63	13.52 ± 4.12	14.83 ± 4.83	0.013
DBIL (μmol/L)	2.30 ± 1.25	2.50 ± 1.35	2.19 ± 1.19	0.037
BMI (kg/m^2^)	24.63 ± 3.16	25.07 ± 3.54	24.39 ± 2.91	0.057
SBP (mmHg)	138.8 ± 19.72	147.7 ± 19.65	134.2 ± 18.14	< 0.001
DBP (mmHg)	77.42 ± 12.45	77.10 ± 12.76	77.59 ± 12.31	0.729
Hypertension (%)	64 (18.4%)	21 (17.5%)	43 (18.9%)	0.669
Type II diabetes (%)	19 (5.5%)	10 (8.3%)	9 (3.9%)	< 0.001
Hyperuricemia (%)	52 (14.9%)	23 (19.2%)	29 (12.7%)	0.038
CysC (mg/L)	1.079 ± 0.64	1.32 ± 0.99	0.95 ± 0.76	< 0.001
TGF-β (pg/mL)	13.23 ± 5.18	17.70 ± 3.22	10.88 ± 4.41	< 0.001
ADMA (μmol/L)	101.1 ± 64.80	118.6 ± 46.47	91.89 ± 70.99	0.004
NGAL (µg/L)	16.55 ± 7.31	14.88 ± 7.72	17.43 ± 6.95	0.087

*FPG* fasting plasma glucose, *TC* total cholesterol, *TG* triglyceride, *BUN* urea nitrogen, *SCr* serum creatinine, *SUA* serum uric acid, *TP* total protein, *ALB* albumin, *GLB* globulin, *ALT* alanine aminotransferase, *TBIL* total bilirubin, *DBIL* direct bilirubin, *BMI* body mass index, *SBP* systolic blood pressure, *DBP* diastolic blood pressure, *CysC* cystatin C, *TGF-β* transforming growth factor-β, *ADMA* asymmetric dimethylarginine, *NGAL* neutrophil gelatinase-associated lipocalin.

Data are expressed as the mean SD, percentage (number), or median (interquartile range); *t* test or Mann–Whitney rank sum test was used for the continuous variables.

### Non-genetic risk factors for CKD

**Figure 2:**
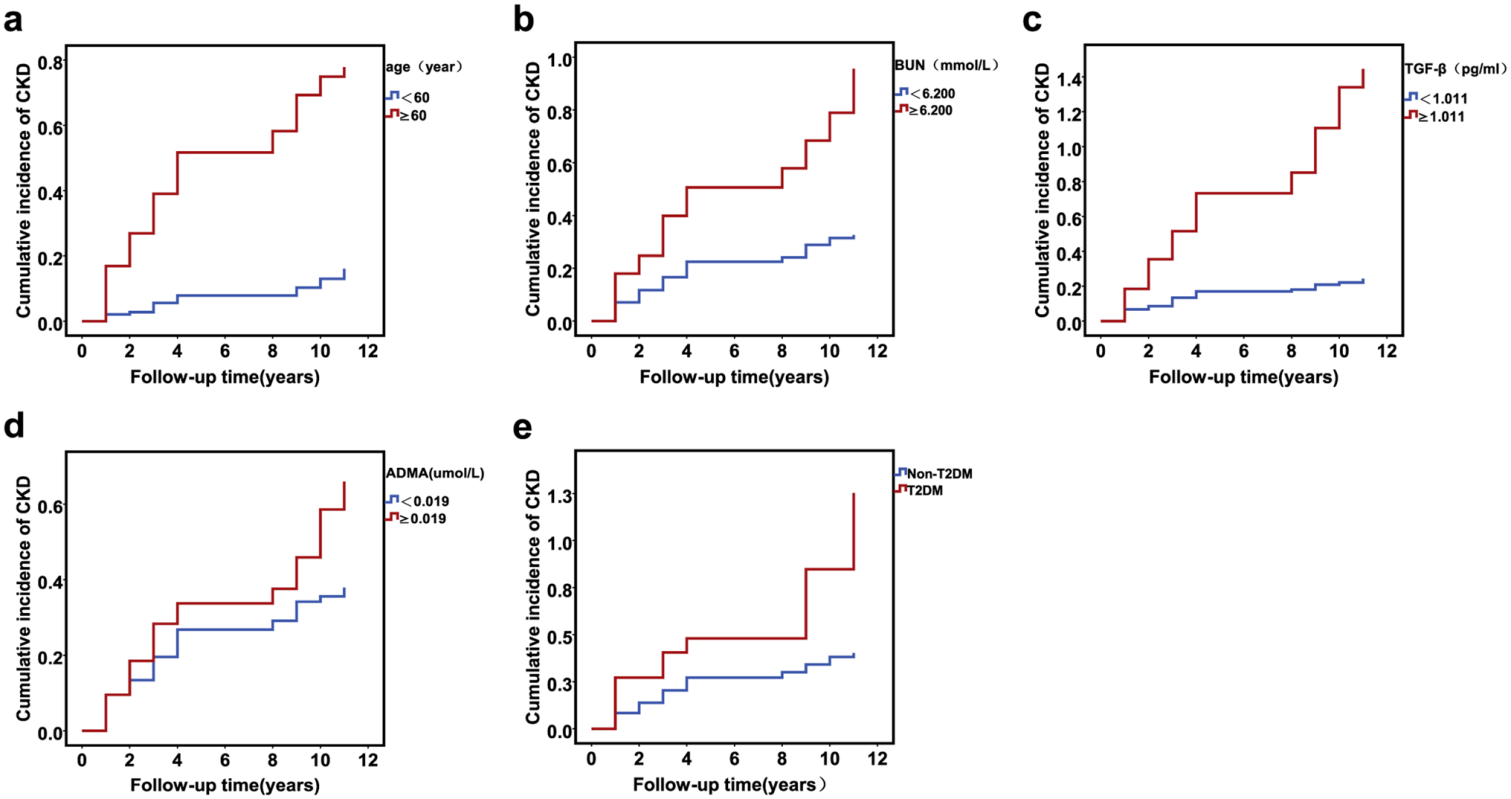
Kaplan–Meier survival curve of CKD cumulative incidence in 348 subjects of the nested case–control study. (**a**) Elderly individuals; (**b**) normal high value of urea nitrogen (BUN); (**c**) normal high value of transforming growth factor-β (TGF-β); (**d**) normal high value of asymmetric dimethylarginine (ADMA); (**e**) diabetes.

A Cox proportional risk regression model showed that age, diabetes mellitus, a normal high value of urea, a normal high value of TGF-β, and ADMA were independent risk factors for CKD (Table 2; Supplementary Table S3). Kaplan–Meier survival analyses showed that the elderly, normal high value of urea nitrogen, normal high value of TGF-β, normal high value of ADMA, and diabetes (we defined age ≥ 60 years as the elderly, taking the higher quartile of other measurement data as their normal high values) were significantly associated with chronic kidney disease onset in our cohort (Fig. 2).

**Table 2:** Non-genetic multivariate Cox regression analyses and non-genetic risk models (NGRS).

Variables	β	SE	χ^2^	HR	95% CI	*P-*value	NGRS model	OR (model)	95% CI (model)	*P-*value (model)
Women	0.216	0.207	1.094	1.241	0.828–1.861	0.296				
Normal high value of TGF-β^a^	0.945	0.195	3.553	2.572	1.756–3.766	< 0.001				
							1. Normal high value of TGF-β, Normal high value of ADMA	3.634	2.723–4.850	< 0.001
Normal high value of ADMA^b^	1.222	0.244	4.999	3.394	2.102–5.479	< 0.001				
							2. Normal high value of TGF-β, Normal high value of ADMA, Diabetes	3.703	2.775–4.942	< 0.001
Diabetes	0.742	0.272	7.450	2.100	1.233–3.578	0.006				
							3. Normal high value of TGF-β, Normal high value of ADMA, Diabetes, Normal high value of BUN	3.917	2.910–5.273	< 0.001
Normal high value of BUN^c^	0.693	0.197	12.335	2.00	1.359–2.946	< 0.001				
							4. Normal high value of TGF-β, Normal high value of ADMA, Diabetes, Normal high value of BUN, The elderly	4.113	3.039–5.566	< 0.001
The elderly^d^	1.055	0.256	16.940	2.872	1.738–4.746	< 0.001				

*TGF-β* transforming growth factor-β, *ADMA* asymmetric dimethylarginine, *BUN* urea nitrogen, *NGRS* non-genetic risk score, *HR* hazard ratio, *CI* confidence interval.

^a^Defined as the serum concentration of TGF-β ≥ 1.011 pg/mL.

^b^Defined as the serum concentration of ADMA ≥ 0.019 μmol/L.

^c^Defined as the serum concentration of BUN ≥ 5.9 mmol/L.

^d^Defined as the age of the participants ≥ 60 years.

### Non-genetic risk score (NGRS) prediction model for CKD

**Figure 3:**
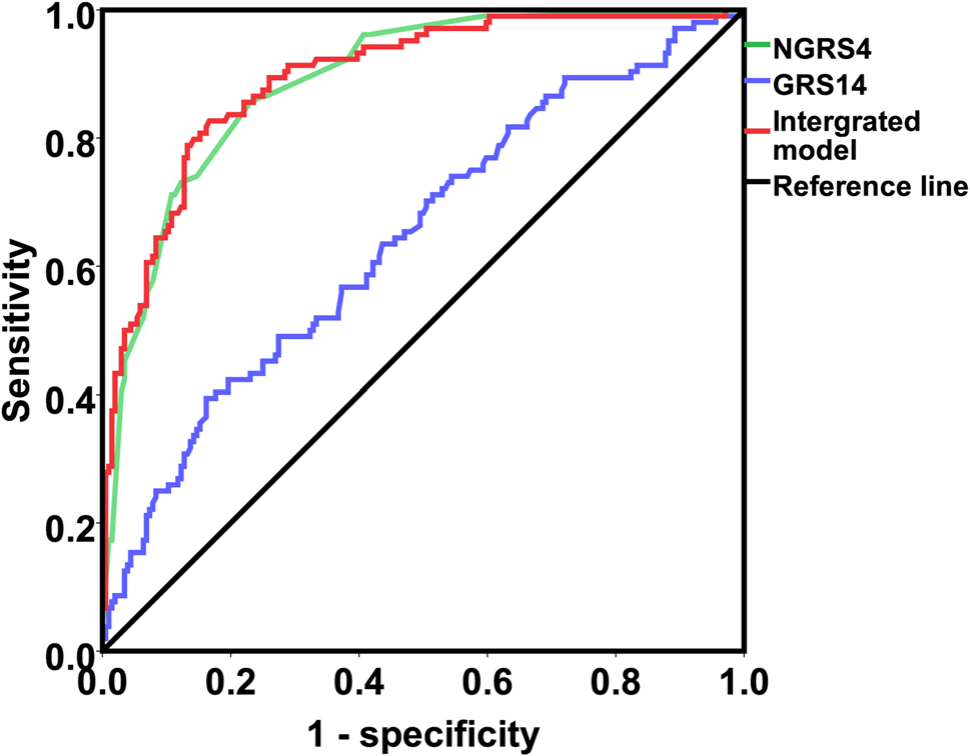
ROC curves of the nongenetic (NGRS4), genetic (GRS14), and comprehensive models for CKD prediction. NGRS4: The No. 4 nongenetic risk score model; GRS14: The No. 14 nongenetic risk score model.

A total of 5 predictors, including age, diabetes mellitus, normal high value of BUN, normal high value of TGF-β, and ADMA, were included in the nongenetic prediction model for CKD. Among the four models (Supplementary Material S1; Table S4; Table S5) that we constructed based on those 5 risk factors, the CKDNGRS4 model yielded the highest C statistic (0.889; 95% CI 0.851–0.925) and the highest OR value (4.113; 95% CI 3.039–5.566) (Table 3; Fig. 3). The prediction equation was logitP = 1.84 × S1 + 1.137 × S2 + 0.84 × S3 + 0.497 × S4 + 0.603 × S5, while S1 = TGF-β normal high value (0: < 1.011 pg/mL; 1: ≥ 1.011 pg/mL), S2 = ADMA normal high value (0: < 0.019 μmol/L; 1: ≥ 0.019 μmol/L), S3 = diabetes (0:unaffected; 1:affected), S4 = BUN normal high value (0: < 5.9 mmol/L; 1: ≥ 5.9 mmol/L), S5 = elderly (0: < 60 years; 1: ≥ 60 years. The sensitivity of the model was 0.851, while the specificity was 0.770.

**Table 3:** Logistic regression analysis and prediction power comparison of nongenetic (NGRS), genetic (GRS), and comprehensive models for CKD.

Models	Logistic regression analysis			ROC curve		
	OR	95%*CI*	*P*-value	AUC	95%*CI*	*P*-value
NGRS4^a^	4.113	3.039–5.566	< 0.001	0.889	0.851–0.925	< 0.001
GRS14^b^	2.363	1.518–3.679	< 0.001	0.643	0.578–0.709	< 0.001
Comprehensive model^c^	3.758	2.827–4.997	< 0.001	0.894	0.857–0.931	< 0.001

*ROC* receiver operating characteristic, *OR* odds ratio, *CI* confidence interval, *AUC* area under curve, *NGRS4* nongenetic risk score model 4, *GRS14* genetic risk score model 14.

^a^NGRS4 = 1.84 × S1 + 1.137 × S2 + 0.84 × S3 + 0.497 × S4 + 0.603 × S5 (S_i_ represents the state of the ith nongenetic risk factor; if the individual has the risk factor, the value is 1; if not, the value is 0. S1 = TGF-β normal high value (0: < 1.011 pg/mL; 1:1.011 pg/mL), S 2 = ADMA normal high value (0: < 0.019 μmol/L; 1: ≥ 0.019 μmol/L), S 3 = diabetes (0:unaffected; 1:affected), S 4 = BUN normal high value (0: < 5.9 mmol/L; 1: ≥ 5.9 mmol/L), S 5 = elderly (0: < 60 years; 1: ≥ 60 years).

^b^GRS14 = 0.577 × rs17319721Gi + (− 0.183) × rs700233Gi + (− 0.362) × rs671Gi + (− 0.286) × rs11864909Gi + 1.099 × rs653178Gi + 0.255 × rs3752462Gi + 0.228 × rs13146355Gi + 0.253 × rs881858Gi + (− 0.24) × rs1153849Gi + (− 0.234) × rs3770636Gi + (− 0.178) × rs504915Gi + 0.149 × rs16853722Gi + 0.683 × rs12917707Gi + (− 0.133) × rs1731274Gi (Gi is the number of alleles at the ith SNP, assigning a value of 0, 1, 2).

^c^Comprehensive model = NGRS4 + GRS14.

### Genetic risk score (GRS) prediction model for CKD

By integrating the results of CKD-related genetic loci in UK Biobank subjects (Supplementary Table S1) and previous studies, 25 SNPs were analyzed for their correlation with CKD by logistic regression analysis (Supplementary Table S7). Seventeen SNPs were selected in the CKD genetic prediction model, including 7 SNPs derived from the UK Biobank. A total of 14 models were constructed (Supplementary Table S8; Material S2). Combining the results of regression analysis and survival analysis (Supplementary Table S9; Table S10), we found that CKDGRS14 was the best prediction model. The area under the ROC curve (AUC) of the model was 0.643 (95% CI 0.578–0.709), the sensitivity of the model was 0.794, and the specificity was 0.838. The OR value was 2.363 (95% CI 1.518–3.679) (Table 3; Fig. 3). The genetic risk prediction equation for CKD was logitP = 0.577 × rs17319721Gi + (− 0.183) × rs700233 + (− 0.362) × rs671Gi + (0.286) × rs11864909Gi + 1.099 × rs653178Gi + 0.255 × rs3752462Gi + 0.228 × rs13146355Gi + 0.253 × rs881858Gi + (− 0.24) × rs1153849Gi + (− 0.234) × rs3770636Gi + (0.178) × rs504915Gi + 0.149 × rs16853722Gi + 0.683 × rs12917707Gi + (− 0.133) × rs1731274Gi.

### Comprehensive prediction model for CKD

Through analysis and screening, CKDNGRS4 and CKDGRS14 were found to be optimal nongenetic predictive models and genetic predictive models, respectively. The final comprehensive predictive model is the arithmetic sum of the two models. It was logitP = 0.577 × rs17319721Gi + (− 0.183) × rs700233 + (− 0.362) × rs671Gi + (− 0.286) × rs11864909Gi + 1.099 × rs653179Gi + 0.255 × rs3752462Gi + 0.228 × rs13146355Gi + 0.253 × rs881858Gi + (− 0.24) × rs1153849Gi + (− 0.234) × rs3770636Gi + (− 0.178) × rs504915Gi + 0.149 × rs16853722Gi + 0.683 × rs12917707Gi + (− 0.133) × rs1731274Gi + 1.84 × S1 + 1.137 × S2 + 0.84 × S3 + 0.497 × S4 + 0.603 × S5. The predictive power of the CKD comprehensive prediction model was higher than that of either of the nongenetic or genetic prediction models: the AUC was 0.894 (95% CI 0.857–0.931), the OR was 3.758 (95% CI 2.827–4.997), the sensitivity was 0.827, and the specificity was 0.801 (Table 3, Fig. 3).

### Internal validation

In the nested case–control study, bootstrap five-fold cross validation was carried out for different prediction models of CKD onset. After the verification results were averaged, the AUC values of the nongenetic, genetic, and comprehensive prediction models of CKD were 0.786, 0.692, and 0.820, respectively.

## Discussion

Early prediction of CKD is challenging. Decades of research have shown that diabetic nephropathy, primary glomerulonephritis, hypertension, interstitial nephritis, and polycystic kidney can all induce CKD. The awareness of CKD is notoriously low; once CKD has developed, treatment is usually limited until the last remedies of dialysis and renal transplantations are needed for ESRD. The eGFR is a sensitive indicator of renal function; however, it is not an early predictor of CKD. Although many biomarkers have been tested for CKD, reappraisal in prospective cohort studies with large sample sizes is needed. Seeking an early, sensitive, easy to perform and cost-effective prediction model.

We carried out a nested case–control study for CKD prediction out of the “Tianjin Medical University Chronic Disease Cohort”^26,27^, with strong pertinence, facilitated prediction of the 5-year probability of chronic kidney disease onset in this area. The average age of the subjects was 63 years; thus, those individuals were more likely to develop CKD than younger subjects.

We combined traditional laboratory indicators, multiple biomarkers related to renal function, and SNP loci to develop CKD prediction models. In the NGRS model, we not only included some indicators that were used in other studies, such as diabetes and age^25,28,29^, but several biomarkers, especially TGF-β and ADMA, were also employed as early CKD predictors in the model.

Although hundreds of associations were found among CKD and susceptibility genes, large sample-sized GWAS also yielded very significant results, and genetic factors only provided a little improvement of the prediction model. Given a certain SNP, the genetic relative risk (GRR) could be high; however, its contribution to CKD risks in the general population was limited. All 17 SNPs employed in our study were from GWASs out of the UK Biobank and other large cohorts; however, the AUC of the genetic risk model (GRS) was only 0.643 and had only given a marginal improvement in the AUC in the comprehensive model (from 0.889 to 0.894). A study in Japan showed that genetic predictors do not contribute significantly to the improvement of the prediction efficiency of the comprehensive prediction model^29^. Although certain SNPs had very significant associations with CKD in large sample-sized GWASs (i.e., high genetic relative risk, GRR), their contribution to phenotype variance might be limited.

Several biomarkers were tested and included in our prediction model. The plasma TGF-β level, alone with ADMA, provided better prediction value than the more direct glomerular filtration indicator cystatin C. In our previous study, we found that TGF-β pathway genes were highly expressed in the kidneys of very early stage diabetic nephropathy renal biopsies, long before renal fibrosis and decreased filtration occurred. Indeed, screening early biomarkers before decreasing eGFR may give CKD predictions several years earlier, although early treatment could be another obstacle to overcome.

This study has a few limitations. First, the research on CKD-related biomarkers was carried out in a nested case–control study that selected from a cohort of chronic diseases, and the sample size was relatively small; therefore, the results from the study may have had certain deviations. Second, our risk prediction model only focused on the onset of chronic kidney disease but did not assess the progression of chronic kidney disease to renal failure or other complications. Third, participants who made up the “Tianjin Medical University Chronic Disease Cohort” were mostly teachers and government employees who worked in urban areas. This group of people were more self-disciplined and paid more attention to health. Whether our prediction model could be applied to other groups of people needs more external validation. Our future studies will detect more renal function-related biomarkers in larger cohorts to validate and improve the prediction model for CKD.

Recently, numerous predictive models have been established and came into use in the clinic for decision-making. Among them, there exist several models estimating the risk of prevalent and incident CKD^22,28–31^. However, due to differences in race, lifestyle, and geographic environment, it is still necessary to develop an effective predictive model for chronic kidney disease in different ethnic groups, which can help to identify people with higher CKD risks earlier, thus improving health care by allocating resources to those individuals who benefit most from it while preventing the potential abuse of health care resources by individuals who are at low risk.

## Methods

### Study design and population

This research was designed as a nested case–control study involving 348 participants from the “Tianjin Medical University Chronic Diseases Cohort”. The cohort was established in 2006, with an initial number of 2068 people for an annual physical examination. By the end of 2018, a total of 21,750 people had been recruited to the cohort, with the longest follow-up period of 13 years. We collected demographic markers, laboratory markers, and genotyping results for 110 loci (including 380 cases with genome-wide genotyping data). We screened patients who met the following criteria: (i) with a follow-up period of at least 5 years; (ii) no CKD at the first physical examination; (iii) blood samples and other important information among whom 1804 were eligible; 116 were selected as the case group; and 232 were selected as the control group with sex and age ± 3 years matching; therefore, a total of 348 subjects were included. All subjects denied family history of inherited diseases and nephrotoxic drug usage.

This study was reviewed and approved by the Ethics Committee of Tianjin Medical University, and all participants signed informed consent forms.

### Diagnostic criteria

The diagnostic criteria for CKD were eGFR < 60 mL/(min·1.73 m^2^) or positive proteinuria (≥ 1 +). The glomerular filtration rate is estimated using the simplified Chinese MDRD equation^32^. The determination of diabetes mellitus (DM) is based on the diagnostic criteria of diabetes published by the World Health Organization (WHO) in 1999: fasting plasma glucose ≥ 7.0 mmol/L and/or 2 h postprandial glucose ≥ 11 mmol/L. Obesity was defined as a body mass index (BMI) ≥ 28 kg/m^2^ according to the recommendation of the “Guidelines for the Prevention and Control of Overweight and Obesity among Chinese Adults”^33^ by the Ministry of Health. Hypertension was defined as systolic blood pressure (SBP) ≥ 140 mmHg and/or diastolic blood pressure (DBP) ≥ 90 mmHg or a self-reported history of physician-diagnosed hypertension. The diagnostic criteria for hyperuricemia (HUA)^34^ were blood uric acid level ≥ 420 μmol/L in men and ≥ 360 μmol/L in women.

### Measurements of biomarkers

After twelve hours of fasting, participants’ venous blood samples were collected into nonanticoagulant blood collection tubes at 7:30–9:00 am, incubated at room temperature for half an hour and then centrifuged at 3000 rpm at 4 °C for 10 min to separate serum. The serum was stored at − 80 °C before analysis. Levels of fasting plasma glucose, serum creatinine, urea nitrogen, serum uric acid, total cholesterol, triglyceride, alanine aminotransferase, total protein, albumin, globulin, total bilirubin, and direct bilirubin were determined using a Hitachi automatic biochemical analyzer. Cystatin C (CysC), transforming growth factor beta (TGF-β), asymmetric dimethylarginine (ADMA) and neutrophil gelatinase-associated lipocalin (NGAL) were measured by ELISA kits (Shanghai Huyu Biotechnology Co., LTD).

### Selection of CKD-related nongenetic/genetic risk factors

We incorporated 21 potential risk factors, including several biomarkers, into the univariate Cox proportional hazard model (Supplementary Table S3), and then significant factors were taken as explanatory variables and incorporated into the multivariate Cox proportional hazard regression model. Finally, we obtained five nongenetic risk factors (Table 2; Fig. 2).

After obtaining part of the data access of the UK-Biobank database, we used PLINK to perform genome-wide association analysis (GWAS) for renal function-related indicators, including eGFR, SCr, and CysC. The results of the GWAS are shown in the Manhattan plot (Supplementary Fig. S1). Combined with the results of previous studies, a total of 10 SNP loci on 10 genes were screened (Supplementary Table S1). Meanwhile, after integrating information from GWAS databases, the UCSC Genomic bioinformatics Database, and GWAS results for kidney function-related phenotypes in Asia or China^35–37^, SNP loci with both high genotype relative risk (GRR) and genome-wide polygenetic score (GPS) for CKD were selected. Finally, we selected a total of 27 SNP loci from 24 genes to construct a genetic risk model for CKD (Supplementary Table S2). The 27 SNPs selected in this study were genotyped in 348 nested case–control subjects using a matrix-assisted laser desorption ionization time-of-flight mass spectrometry (MALDI-TOF–MS) platform. Hardy–Weinberg equilibrium (HWE) was checked for all 27 SNPs, and we deleted 2 SNPs that failed HWE; therefore, genotyping data for 25 SNPs were documented.

### Developing prediction models

In this study, genetic risk score (GRS) models and nongenetic risk score (NGRS) models were built from the weights of natural logarithms (β) of different risk factors’ OR values. The combined effects of each nongenetic or genetic factor were calculated in a weighted way, and the optimal combination method was selected to develop the prediction model of CKD. The GRS equation was established based on the different contributions of each candidate SNP site to the pathogenesis of CKD. Each SNP site was considered a potential risk factor for CKD. Different weights for the contribution to the onset of CKD were determined by different OR (or β) values from logistic regression analysis to establish several combinations and screen for the optimal combination. Using a weighted genetic risk score (ωGRS), ωGRS = $\sum\nolimits_{1}^{i} {\upbeta_{{\rm i}} } {\text{G}}_{{\rm i}}$ (β_i_ is the weight of the ith SNP, G_i_ is the number of alleles at the ith SNP, and assigns a value of 0, 1, 2). The weight is the natural logarithm of the odds ratio (OR) of SNPs and could be an estimated effect (β coefficient). For each individual, ωGRS is the sum of the number of risk alleles weighted by the OR (β) value of each SNP site in logistic regression. See Formula (1) for details.

1 $$\begin{aligned} {\text{GRS}} &= \sum\limits_{1}^{{\rm i}} {\upbeta_{{{\rm i}}} {\text{G}}_{{{\rm i}}} }\\ {\text{Logit}}P &= \upalpha + \upbeta ( {{\text{GRS}}} )\\ &= \upalpha + \upbeta \sum\limits_{1}^{i} {\upbeta_{{{\rm i}}} {\text{G}}_{{{\rm i}}} } \end{aligned}$$

In the above formula, to fix the weight in advance, we used the value of log-converted single-risk alleles in studies with large sample sizes and high reliability (e.g., meta-analysis) as the weight in the actual model construction.

The building principle of the nongenetic risk score model is the same as that of the GRS. That is, according to the different contributions of the identified CKD-related nongenetic risk factors (e.g., normal high value of TGF-β, the elderly) to the incidence of CKD, different OR (or β) values of logistic regression analysis are used to determine different weights for the onset of CKD, establish different combinations and select the optimal combination. The weighted nongenetic risk score (ωNGRS) was used, ωNGRS = $\sum\nolimits_{1}^{i} {\upbeta_{{\rm i}} } {\text{S}}_{{\rm i}}$ (β_i_ is the weight of the ith corresponding nongenetic risk factor in the risk of developing CKD, and S_i_ is the ith corresponding nongenetic risk factor), and the weight β takes the natural logarithm of the OR value obtained by logistic regression analysis of different risk factors. For every individual, ωNGRS is the sum of risk factors weighted by the OR (β) value of different nongenetic risk factors in logistic regression. See Formula (2) for details.

2 $$\begin{aligned} {\text{NGRS}} &= \sum\limits_{1}^{{\rm i}} {\upbeta_{{{\rm i}}} {\text{S}}_{{{\rm i}}} }\\ {\text{Logit}}P &= \upalpha + \upbeta ( {{\text{NGRS}}} )\\ &= \upalpha + \upbeta \sum\limits_{1}^{{\rm i}} {\upbeta_{{{\rm i}}} {\text{S}}_{{{\rm i}}} } \end{aligned}$$

In the above formula, S represents the set vector of a group of nongenetic risk factors (S_i_ represents the state of the ith nongenetic risk factor; if the individual has the risk factor, the value is 1; if not, the value is 0). The β value used in this study was the β value of each nongenetic risk factor in logistic regression analysis.

The construction of the comprehensive risk scoring model integrates the optimal GRS model and the NGRS model, which is the sum of the two models. See formula (3) for details.

3 $$\begin{aligned} {\text{Logit}}P &= \upalpha + \upbeta ({\text{GRS}} + {\text{NGRS}})\\ &= \upalpha + \upbeta \left( {\sum\limits_{1}^{{\rm i}} {\upbeta_{{{\rm i}}} {\text{G}}_{{{\rm i}}} } + \sum\limits_{1}^{i} {\upbeta_{{{\rm i}}} {\text{S}}_{{{\rm i}}} } } \right) \end{aligned}$$

### Prediction model evaluation

The evaluation of the constructed GRS model, NGRS model and comprehensive predictive model adopted the receiver operating characteristic curve (ROC) area under the curve (AUC) method. MedCalc software was used to determine the optimal cut-off point of the ROC curve and the sensitivity and specificity at the optimal cut-off point. Finally, the evaluation of the prediction effectiveness of the constructed CKD prediction model is realized. The constructed GRS model, NGRS model and comprehensive prediction model were internally validated in a nesting case–control study using bootstrap five-fold cross-validation. All data analyses were performed using SPSS 21.0 software. Statistical significance was determined with a threshold *P* value of < 0.05.

All methods were performed in accordance with the relevant guidelines and regulations.

## Conclusion

Age, diabetes, normal high values of creatinine, TGF-β, and ADMA are independent indicators for CKD incidence. A comprehensive prediction model was established, although genetic factors that analyzed in our study yielded limited prediction values for CKD incidence. Early and appropriate intervention can be exerted to avoid getting worse and even irreversible.

## Supplementary Information

Supplementary Information.
