# Supplementary material for: An early prediction model for chronic kidney disease
Source: Sci Rep. 2022 Feb 17;12:2765. doi: 10.1038/s41598-022-06665-y (PMC8854510; doi:10.1038/s41598-022-06665-y)
Supplement: Supplementary file 1 — Supplementary Information. [file 41598_2022_6665_MOESM1_ESM.pdf]

Supplementary material

Table S1. Single nucleotide polymorphisms (SNPs) that associated with renal function related phenotypes in UK-biobank subjects

Table S2. CKD candidate gene loci that selected for genetic risk models

Table S3. Univariate Cox regression analyses for non-genetic risk factors

Material S1. Four CKD NGRS models that constructed with 5 selected non-genetic risk factors

Table S4. Logistic regression analyses for different CKD NGRS models

Table S5. Comparison of predictive powers of different CKD NGRS models

Table S6. Hardy-Weinberg Equilibrium tests for 27 CKD candidate gene loci

Table S7. Logistic regression analyses of CKD related candidate gene SNPs

Table S8. GRS models including different SNP loci that related to CKD

Material S2. GRS models including different SNPs that related to CKD

Table S9. Logistic regression analyses for GRS models

Table S10. Comparison of predictive powers of different GRS models

Figure S1. Manhattan map for genome-wide association analysis of CysC, SCr, and eGFR in UK Biobank subjects

Table S1. Single nucleotide polymorphisms (SNPs) that associated with renal function related phenotypes in UK-biobank subjects

SNP	Gene	eGFR(*P*-value)	SCr(*P*-value)	CysC(*P*-value)
rs13146355	*SHROOM3*	1.308×10^{-189}	2.55×10^{-99}	7.41×10^{-266}
rs7219624	*BCAS3*		2.57×10^{-148}	6.13×10^{-47}
rs10224002	*PRKAG2*	7.24×10^{-263}	2.15×10^{-156}	3.84×10^{-84}
rs653178	*SH2B3(ATXN2)*			3.49×10^{-239}
rs1153849	*GATM*	7.24×10^{-263}	2.00×10^{-226}	3.11×10^{-28}
rs12917707	*UMOD*	8.10×10^{-162}	1.50×10^{-115}	4.00×10^{-58}
rs316019	*SLC22A2*	4.61×10^{-71}	1.17×10^{-41}	
rs3850625	*CACNC1S*	2.26×10^{-55}	2.60×10^{-31}	
rs267738	*CERS2*	3.26×10^{-58}	1.12×10^{-44}	
rs700233	*C9*	2.05×10^{-83}	1.32×10^{-49}	3.00×10^{-30}

Table S2. CKD candidate gene loci that selected for genetic risk models

Number	SNP	Gene	Chr	Base-pair position	MAF(CHB)
1	rs10277115	*UNCX*	7	1245559	0.34(T)
2	rs11864909	*UMOD*	16	20389517	0.14(T)
3	rs16853722	*MECOM*	3	169432844	0.34(C)
4	rs17319721	*SHROOM3*	4	76447694	0.13(A)
5	rs17730281	*WDR72*	15	53615751	0.50(A)
6	rs13146355	*SHROOM3*	4	76490987	0.13(A)
7	rs2390793	*LRP2*	2	169348673	0.19(T)
8	rs3770636	*LRP2*	2	169346323	0.22(G)
9	rs4744712	*PIP5K1B*	9	68819791	0.43(A)
10	rs504915	*SLC22A12*	11	64696613	0.29(A)
11	rs881858	*VEGFA*	6	43838872	0.18(G)
12	rs889472	*MAF*	16	79612092	0.38(A)
13	rs7219624	*BCAS3*	17	61382803	0.24(G)
14	rs671	*ALDH2*	12	111803962	0.15(A)
15	rs3752462	*MYH*	22	036314138	0.30(C)
16	rs2231142	*ABCG2*	4	88131171	0.28(T)
17	rs10224002	*PRKAG2*	7	151717955	0.06(G)
18	rs653178	*SH2B3(ATXN2)*	12	111569952	0.19(T)
19	rs1153849	*GATM*	15	45403497	0.81(A)
20	rs12917707	*UMOD*	16	20356368	0.13(T)

21	rs316019	*SLC22A2*	6	160249250	0.86(C)
22	rs3850625	*CACNCA1S*	1	201047168	0.04(A)
23	rs113956264	*RPL3L*	16	1947003	0.03(T)
24	rs267738	*CERS2*	1	150968149	0.03(G)
25	rs700233	*C9*	5	39364452	0.07(A)
26	rs1731274	*STC1*	8	23908806	0.71(A)
27	rs13038305	*CST*	20	23629625	0.12(T)

CKD related gene loci were selected base on:(1) With reference to Chinese genotype frequencies, SNP loci with minimum allele frequencies greater than 5% were selected; (2) Consider the physical location distribution of SNP loci in genes. Focus on known biologically significant mutations such as missense mutations in the coding region of the gene; (3) They were significantly associated with CKD in other studies.

Table S3. Univariate Cox regression analyses for non-genetic risk factors

Characteristics	β	SE	χ^2	RR	95%*CI*	*P-value*
Age, year						
<50	1(ref.)			1(ref.)		
50-59	-0.055	0.563	0.009	0.947	0.314-2.852	0.992
60-69	0.798	0.529	2.276	2.220	0.788-6.257	0.031
70-79	1.943	0.522	13.829	6.979	2.507-19.43	0.000
80-100	2.961	0.550	28.997	19.314	6.574-26.74	0.000
Age as a continuous variable	0.110	0.011	15.163	1.116	1.093-1.140	0.000
Sex						
Male	1(ref.)			1(ref.)		
Female	0.244	0.199	1.499	1.276	0.864-1.886	0.221
Type II diabetes						
Without	1(ref.)			1(ref.)		
With	0.486	0.330	2.168	1.626	0.851-3.108	0.041
FPG as a continuous variable	0.168	0.073	5.325	1.183	1.026-1.364	0.021
TC(mmol/L)						
<4.4	1(ref.)			1(ref.)		
4.4-5.0	0.139	0.288	0.233	1.149	0.654-2.021	0.629
5.1-5.5	0.316	0.297	1.131	1.372	0.766-2.457	0.288
>5.5	0.688	0.279	6.086	1.989	1.152-3.400	0.121
TC as a continuous variable	0.248	0.097	6.586	1.282	1.060-1.550	0.010
TG(mmol/L)						
<1.05	1(ref.)			1(ref.)		
1.05-1.45	-0.337	0.266	1.600	0.714	0.424-1.203	0.206
1.46-2.08	0.024	0.250	0.009	1.024	0.627-1.672	0.924
>2.08	-0.117	0.256	0.209	0.889	0.538-1.470	0.648
TG as a continuous variable	0.034	0.064	0.284	1.035	0.913-1.173	0.594
Hyperuricemia						

Without	1(ref.)					
With	0.378	0.232	2.657	1.46	0.926-2.300	0.103
BUN(mmol/L)						
<4.7	1(ref.)			1(ref.)		
4.7-5.35	0.747	0.358	4.356	2.110	1.047-4.254	2.773
5.36-6.1	1.020	0.348	8.568	2.773	1.401-5.488	0.501
>6.1	1.714	0.334	26.284	5.552	2.883-10.69	0.000
BUN as a continuous variable	0.437	0.077	32.131	1.547	1.331-1.799	0.000
SCr(μmmol/L)						
<74	1(ref.)			1(ref.)		
74-83	0.562	0.308	3.337	1.755	0.960-3.209	0.068
83.1-93	0.284	0.335	0.715	1.328	0.688-2.563	0.398
>93	1.594	0.287	30.924	4.925	2.808-8.638	0.000
SCr as a continuous variable	0.051	0.008	46.272	1.053	1.037-1.069	0.000
SUA(μmmol/L)						
<281	1(ref.)			1(ref.)		
281-331	0.289	0.27	1.141	1.335	0.786-2.662	0.285
331.1-383.75	0.317	0.276	1.318	1.373	0.799-2.358	0.251
>383.75	0.469	0.265	3.123	1.598	0.950-2.687	0.027
SUA as a continuous variable	0.002	0.001	3.027	1.002	1.000-1.004	0.082
TP(g/L)						
<73	1(ref.)			1(ref.)		
73-75	-0.168	0.263	0.406	0.845	0.505-1.417	0.524
75.1-78	0.008	0.261	0.001	1.008	0.604-1.683	0.974
>78	0.261	0.265	0.965	1.298	0.772-2.183	0.326
TP as a continuous variable	0.031	0.022	2.072	1.032	0.989-1.077	0.150
ALB(g/L)						
<44	1(ref.)			1(ref.)		
44-46	0.097	0.259	0.142	1.102	0.664-1.830	0.707
46.1-47	0.273	0.299	0.836	1.314	0.732-2.361	0.360

>47	-0.175	0.302	0.335	0.840	0.465-1.517	0.563
ALB as a continuous variable	-0.035	0.035	0.988	0.965	0.901-1.035	0.320
GLB(g/L)						
<27	1(ref.)			1(ref.)		
27-29	-0.262	0.294	0.797	0.769	0.432-1.368	0.372
29.1-32	-0.081	0.312	0.067	0.922	0.501-1.699	0.796
>32	0.463	0.305	2.306	1.589	0.874-2.887	0.129
GLB as a continuous variable	0.067	0.026	6.550	1.069	1.016-1.125	0.010
ALI(IU/L)						
<20	1(ref.)			1(ref.)		
20-24	-0.428	0.248	2.974	0.652	0.400-1.060	0.085
24.1-32	-0.307	0.247	1.555	0.735	0.454-1.192	0.212
>32	-0.650	0.281	5.348	0.522	0.301-0.906	0.021
ALT as a continuous variable	-0.031	0.012	7.117	0.969	0.947-0.992	0.008
TBIL(μmol/L)						
<11	1(ref.)			1(ref.)		
11-14.2	-0.382	0.240	2.529	0.683	0.426-1.093	0.112
14.3-17.5	-0.479	0.255	3.542	0.619	0.376-1.020	0.060
>17.5	-0.549	0.261	4.428	0.578	0.346-0.963	0.035
TBIL as a continuous variable	-0.051	0.022	5.405	0.950	0.911-0.992	0.02
DBIL(μmol/L)						
<1.4	1(ref.)			1(ref.)		
1.4-2.1	-0.145	0.272	0.283	0.865	0.507-1.475	0.595
2.2-3.0	0.064	0.265	0.058	1.066	0.634-1.794	0.809
>3.0	0.492	0.255	3.730	1.636	0.993-2.697	0.053
TBIL as a continuous variable	0.200	0.071	7.868	1.221	1.062-1.405	0.005
Hypertension						
Without	1(ref.)			1(ref.)		
With	-0.138	0.240	0.329	0.871	0.544-1.395	0.566

Obesity						
Without	1(ref.)			1(ref.)		
With	0.220	0.183	1.444	1.245	0.871-1.782	0.230
CysC(mg/L)						
<0.78	1(ref.)			1(ref.)		
0.78-1.0	0.786	0.340	5.347	2.194	1.127-4.271	0.021
1.01-1.32	1.100	0.325	11.453	3.003	1.588-5.677	0.001
>1.32	1.401	0.314	19.852	4.058	2.192-7.516	0.000
CysC as a continuous variable	0.008	0.003	7.262	1.008	1.002-1.014	0.007
TGF-β(pg/mL)						
<8.36	1(ref.)			1(ref.)		
8.36-12.84	1.748	0.769	5.170	5.743	1.273-5.916	0.023
12.85-18.42	3.331	0.724	21.194	27.978	6.774-9.553	0.000
>18.42	3.877	0.719	29.089	48.282	9.801-17.548	0.000
TGF-β as a continuous variable	0.235	0.024	17.606	1.265	1.207-1.325	0.000
NGAL(μmol/L)						
<12.34	1(ref.)			1(ref.)		
12.34-14.96	-0.911	0.239	14.524	0.402	0.252-0.642	0.000
14.97-18.04	-1.358	0.273	24.769	0.257	0.151-0.439	0.000
>18.04	-1.116	0.254	19.397	0.327	0.199-0.538	0.000
NGAL as a continuous variable	-0.057	0.019	9.222	0.945	0.911-0.980	0.002
ADMA(μg/L)						
<60.04	1(ref.)			1(ref.)		
60.04-89.86	1.650	0.550	8.991	5.206	1.771-5.307	0.003
89.87-122.06	3.172	0.518	17.570	23.866	8.654-10.816	0.000
>122.06	2.456	0.527	21.730	11.655	4.151-9.730	0.000
ADMA as a continuous variable	0.003	0.001	12.691	1.003	1.002-1.005	0.000

Material S1. Four CKDNGRS models that constructed with 5 selected non-genetic risk factors

$$\mathrm{CKDNGRS1}=1.84\times \mathrm{S1}+1.137\times \mathrm{S2} \tag{1}$$

$$\mathrm{CKDNGRS2}=1.84\times \mathrm{S1}+1.137\times \mathrm{S2}+0.84\times \mathrm{S3} \tag{2}$$

$$\mathrm{CKDNGRS3}=1.84\times \mathrm{S1}+1.137\times \mathrm{S2}+0.84\times \mathrm{S3}+0.497\times \mathrm{S4} \tag{3}$$

$$\mathrm{CKDNGRS4}=1.84\times \mathrm{S1}+1.137\times \mathrm{S2}+0.84\times \mathrm{S3}+0.497\times \mathrm{S4}+0.603\times \mathrm{S5} \tag{4}$$

In the formula above, S1=Normal high value of TGF-β(0: <1.011pg/ml; 1: ≥ 1.011pg/ml), S2=Normal high value of ADMA(0: <0.019μmol/L; 1: ≥ 0.019μmol/L), S3=Diabetes(0: with; 1: without), S4=Normal high value of BUN(0: <5.9mmol/L; 1: ≥ 5.9mmol/L), S5= The elderly(0: <60years; 1: ≥ 60years).

Table S4. Logistic regression analyses for different CKD NGRS models

NGRS model	OR	95%*CI*	*P*-value
NGRS1	3.634	2.723-4.850	<0.001
NGRS2	3.703	2.775-4.942	<0.001
NGRS3	3.917	2.910-5.273	<0.001
NGRS4	4.113	3.039-5.566	<0.001

Table S5. Comparison of predictive powers of different CKD NGRS models

NGRS model	AUC	95%*CI*	*P*-value
NGRS1	0.831	0.782-0.879	<0.001
NGRS2	0.841	0.794-0.888	<0.001
NGRS3	0.865	0.822-0.907	<0.001
NGRS4	0.889	0.851-0.925	<0.001

Table S6. Hardy-Weinberg Equilibrium tests for 27 CKD candidate gene loci

No.	SNP	Gene	Detectable rate (%)	HWE (*P*-value)	HWE (*P*-value) case	HWE (*P*-value) control
1	rs10277115	*UNCX*	99	0.9057	0.6799	0.6623
2	rs11864909	*UMOD*	100	0.1424	0.6769	0.1526
3	rs16853722	*MECOM*	100	0.8961	0.6682	1
4	rs17319721	*SHROOM3*	100	0.265	0.2798	0.6854
5	rs17730281	*WDR72*	100	0.8107	1	0.7659
6	rs13146355	*SHROOM3*	100	0.6063	0.7908	0.3722
7	rs2390793	*LRP2*	100	0.1469	0.1907	0.3899
8	rs3770636	*LRP2*	100	0.1548	0.1581	0.4098
9	rs4744712	*PIP5K1B*	100	0.01657[a]	0.07832	0.1223
10	rs504915	*SLC22A12*	100	1	0.5841	0.6028
11	rs881858	*VEGFA*	100	0.5277	0.7422	0.2659
12	rs889472	*MAF*	100	0.02745[b]	0.843	0.01043[c]
13	rs7219624	*BCAS3*	100	1	0.5978	0.7252
14	rs671	*ALDH2*	100	0.2466	1	0.1922
15	rs3752462	*MYH9*	100	0.3728	0.8094	0.1806
16	rs2231142	*ABCG2*	100	0.6961	0.252	0.752
17	rs10224002	*PRKAG2*	100	1	1	1
18	rs653178	*SH2B3(ATXN2)*	100	1	1	1
19	rs1153849	*GATM*	100	0.1278	1	0.1189
20	rs12917707	*UMOD*	99	1	1	1
21	rs316019	*SLC22A2*	100	1	1	1
22	rs3850625	*CACNCA1S*	100	1	1	1
23	rs113956264	*RPL3L*	100	1	1	1
24	rs267738	*CERS2*	100	1	1	1
25	rs700233	*C9*	100	0.4888	0.4642	0.2261
26	rs1731274	*STC1*	99	1	0.7763	1
27	rs13038305	*CST*	100	0.4426	0.6876	0.7468

[a,b,c]$P<0.05$, therefore the two SNPs (rs4744712 and rs889472) didn't pass Hardy-Weinberg equilibrium test.

Table S7. Logistic regression analyses of CKD related candidate gene SNPs

No.	SNP	β	SE	χ^2	OR	95%*CI*	*P*-value	*P*-value ranking
1	rs10277115	-0.033	0.174	0.035	0.968	0.688-1.362	0.851	22
2	rs11864909	-0.286	0.228	1.567	0.752	0.481-1.175	0.211	4
3	rs16853722	0.147	0.181	0.664	1.159	0.813-1.652	0.415	12
4	rs17319721	0.577	0.251	5.288	1.781	1.089-2.913	0.021	1
5	rs17730281	-0.012	0.176	0.004	0.988	0.700-1.396	0.947	24
6	rs13146355	0.228	0.203	1.255	1.256	0.843-1.870	0.263	7
7	rs2390793	-0.155	0.243	0.409	0.856	0.532-1.378	0.522	15
8	rs3770636	-0.234	0.246	0.907	0.791	0.489-1.281	0.341	10
9	rs504915	-0.178	0.196	0.822	0.837	0.570-1.229	0.365	11
10	rs881858	0.253	0.233	1.175	1.288	0.815-2.034	0.278	8
11	rs7219624	0.026	0.267	0.010	1.026	0.608-1.734	0.922	23
12	rs671	-0.362	0.247	2.154	0.696	0.429-1.129	0.142	3
13	rs3752462	0.225	0.199	1.281	1.252	0.848-1.849	0.258	6
14	rs2231142	-0.074	0.185	0.163	0.928	0.646-1.333	0.687	19
15	rs10224002	0.182	0.387	0.222	1.200	0.562-2.561	0.637	18
16	rs653178	1.099	0.921	1.423	3.000	0.493-18.24	0.233	5
17	rs1153849	-0.240	0.234	1.048	0.787	0.497-1.245	0.306	9
18	rs12917707	0.683	1.007	0.460	1.980	0.275-14.26	0.498	13
19	rs316019	0.053	0.272	0.038	1.054	0.619-1.795	0.845	21
20	rs3850625	-0.247	0.423	0.343	0.781	0.341-1.787	0.558	17
21	rs113956264	0.298	0.479	0.389	1.348	0.527-3.445	0.533	16
22	rs267738	-0.019	0.432	0.002	0.981	0.421-2.287	0.964	25
23	rs700233	-0.183	0.313	0.340	0.833	0.451-1.539	0.056	2
24	rs1731274	-0.133	0.202	0.434	0.876	0.590-1.300	0.510	14
25	rs13038305	0.091	0.261	0.121	1.095	0.657-1.824	0.728	20

Table S8. GRS models including different SNP loci that related to CKD

GRS model	Included SNP loci number
CKDGRS4	4,23,12,2
CKDGRS5	4,23,12,2,16
CKDGRS6	4,23,12,2,16,13
CKDGRS7	4,23,12,2,16,13,6
CKDGRS8	4,23,12,2,16,13,6,10
CKDGRS9	4,23,12,2,16,13,6,10,17
CKDGRS10	4,23,12,2,16,13,6,10,17,8
CKDGRS11	4,23,12,2,16,13,6,10,17,8,9
CKDGRS12	4,23,12,2,16,13,6,10,17,8,9,3
CKDGRS13	4,23,12,2,16,13,6,10,17,8,9,3,18
CKDGRS14	4,23,12,2,16,13,6,10,17,8,9,3,18,24
CKDGRS15	4,23,12,2,16,13,6,10,17,8,9,3,18,24,7
CKDGRS16	4,23,12,2,16,13,6,10,17,8,9,3,18,24,7,21
CKDGRS17	4,23,12,2,16,13,6,10,17,8,9,3,18,24,7,21,20

Material S2. GRS models including different SNPs that related to CKD

CKDGRS4=0.577×rs17319721Gi+(-0.183)×rs700233Gi+(-0.362)×rs671Gi+(-0.286)×rs11864909Gi

CKDGRS5=0.577×rs17319721Gi+(-0.183)×rs700233Gi+(-0.362)×rs671Gi+(-0.286)×rs11864909Gi+1.099×rs653178Gi

CKDGRS6=0.577×rs17319721Gi+(-0.183)×rs700233Gi+(-0.362)×rs671Gi+(-0.286)×rs11864909Gi+1.099×rs653178Gi+0.255×rs3752462Gi

CKDGRS7=0.577×rs17319721Gi+(-0.183)×rs700233Gi+(-0.362)×rs671Gi+(-0.286)×rs11864909Gi+1.099×rs653178Gi+0.255×rs3752462Gi+0.228×rs13146355Gi

CKDGRS8=0.577×rs17319721Gi+(-0.183)×rs700233Gi+(-0.362)×rs671Gi+(-0.286)×rs11864909Gi+1.099×rs653178Gi+0.255×rs3752462Gi+0.228×rs13146355Gi+0.253×rs881858Gi

CKDGRS9=0.577×rs17319721Gi+(-0.183)×rs700233Gi+(-0.362)×rs671Gi+(-0.286)×rs11864909Gi+1.099×rs653178Gi+0.255×rs3752462Gi+0.228×rs13146355Gi+0.253×rs881858Gi+(-0.24)×rs1153849Gi

CKDGRS10=0.577×rs17319721Gi+(-0.183)×rs700233Gi+(-0.362)×rs671Gi+(-0.286)×rs11864909Gi+1.099×rs653178Gi+0.255×rs3752462Gi+0.228×rs13146355Gi+0.253×rs881858Gi+(-0.24)×rs1153849Gi+(-0.234)×3770636Gi

CKDGRS11=0.577×rs17319721Gi+(-0.183)×rs700233Gi+(-0.362)×rs671Gi+(-0.286)×rs11864909Gi+1.099×rs653178Gi+0.255×rs3752462Gi+0.228×rs13146355Gi+0.253×rs881858Gi+(-0.24)×rs1153849Gi+(-0.234)×3770636Gi+(-0.178)×rs504915Gi

CKDGRS12=0.577×rs17319721Gi+(-0.183)×rs700233Gi+(-0.362)×rs671Gi+(-0.286)×rs11864909Gi+1.099×rs653178Gi+0.255×rs3752462Gi+0.228×rs13146355Gi+0.253×rs881858Gi+(-0.24)×rs1153849Gi+(-0.234)×3770636Gi+(-0.178)×rs504915Gi+0.149×rs16853722Gi

CKDGRS13=0.577×rs17319721Gi+(-0.183)×rs700233Gi+(-0.362)×rs671Gi+(-0.286)×rs11864909Gi+1.099×rs653178Gi+0.255×rs3752462Gi+0.228×rs13146355Gi+0.253×rs881858Gi+(-0.24)×rs1153849Gi+(-0.234)×3770636Gi+(-0.178)×rs504915Gi+0.149×rs16853722Gi+0.683×rs12917707Gi

CKDGRS14=0.577×rs17319721Gi+(-0.183)×rs700233Gi+(-0.362)×rs671Gi+(-0.286)×rs11864909Gi+1.099×rs653178Gi+0.255×rs3752462Gi+0.228×rs13146355Gi+0.253×rs881858Gi+(-0.24)×rs1153849Gi+(-0.234)×3770636Gi+(-0.178)×rs504915Gi+0.149×rs16853722Gi+0.683×rs12917707Gi+(-0.133)×rs1731274Gi

CKDGRS15=0.577×rs17319721Gi+(-0.183)×rs700233Gi+(-0.362)×rs671Gi+(-0.286)×rs11864909Gi+1.099×rs653178Gi+0.255×rs3752462Gi+0.228×rs13146355Gi+0.253×rs881858Gi+(-0.24)×rs1153849Gi+(-0.234)×3770636Gi+(-0.178)×rs504915Gi+0.149×rs16853722Gi+0.683×rs12917707Gi+(-0.133)×rs1731274Gi+(-0.155)×rs2390793Gi

CKDGRS16=0.577×rs17319721Gi+(-0.183)×rs700233Gi+(-0.362)×rs671Gi+(-0.286)×rs11864909Gi+1.099×rs653178Gi+0.255×rs3752462Gi+0.228×rs13146355Gi+0.253×rs881858Gi+(-0.24)×rs1153849Gi+(-0.234)×3770636Gi+(-0.178)×rs504915Gi+0.149×rs16853722Gi+0.683×rs12917707Gi+(-0.133)×rs1731274Gi+(-0.155)×rs2390793Gi+0.298×rs113956264Gi

CKDGRS17=0.577×rs17319721Gi+(-0.183)×rs700233Gi+(-0.362)×rs671Gi+(-0.286)×rs11864909Gi+1.099×rs653178Gi+0.255×rs3752462Gi+0.228×rs13146355Gi+0.253×rs881858Gi+(-0.24)×rs1153849Gi+(-0.234)×3770636Gi+(-0.178)×rs504915Gi+0.149×rs16853722Gi+0.683×rs12917707Gi+(-0.133)×rs1731274Gi+(-0.155)×rs2390793Gi+0.298×rs113956264Gi+(-0.247)×rs3850625Gi

Table S9. Logistic regression analyses for GRS models

GRS model	OR	95%*CI*	*P*-value
CKDGRS4	2.785	1.429-5.427	0.003
CKDGRS5	2.760	1.486-5.126	0.001
CKDGRS6	2.703	1.508-4.846	0.001
CKDGRS7	2.252	1.371-2.698	0.001
CKDGRS8	2.272	1.403-3.680	0.001
CKDGRS9	2.331	1.447-3.752	0.000
CKDGRS10	2.340	1.468-3.731	0.000
CKDGRS11	2.368	1.493-3.757	0.000
CKDGRS12	2.422	1.530-3.834	0.000
CKDGRS13	2.409	1.534-3.785	0.000
CKDGRS14	2.363	1.518-3.679	0.000
CKDGRS15	2.263	1.477-3.466	0.000
CKDGRS16	2.324	1.515-3.566	0.000
CKDGRS17	2.334	1.521-3.581	0.000

Table S10. Comparison of predictive powers of different GRS models

GRS model	AUC	95%*CI*	*P*-value
CKDGRS4	0.593	0.526-0.661	0.007
CKDGRS5	0.594	0.526-0.662	0.007
CKDGRS6	0.603	0.534-0.673	0.003
CKDGRS7	0.589	0.521-0.658	0.010
CKDGRS8	0.599	0.531-0.667	0.004
CKDGRS9	0.613	0.546-0.680	0.001
CKDGRS10	0.621	0.555-0.687	0.001
CKDGRS11	0.628	0.562-0.695	0.000
CKDGRS12	0.633	0.567-0.699	0.000
CKDGRS13	0.638	0.572-0.704	0.000
CKDGRS14	0.643	0.578-0.709	0.000
CKDGRS15	0.640	0.575-0.706	0.000
CKDGRS16	0.637	0.571-0.703	0.000
CKDGRS17	0.642	0.576-0.707	0.000

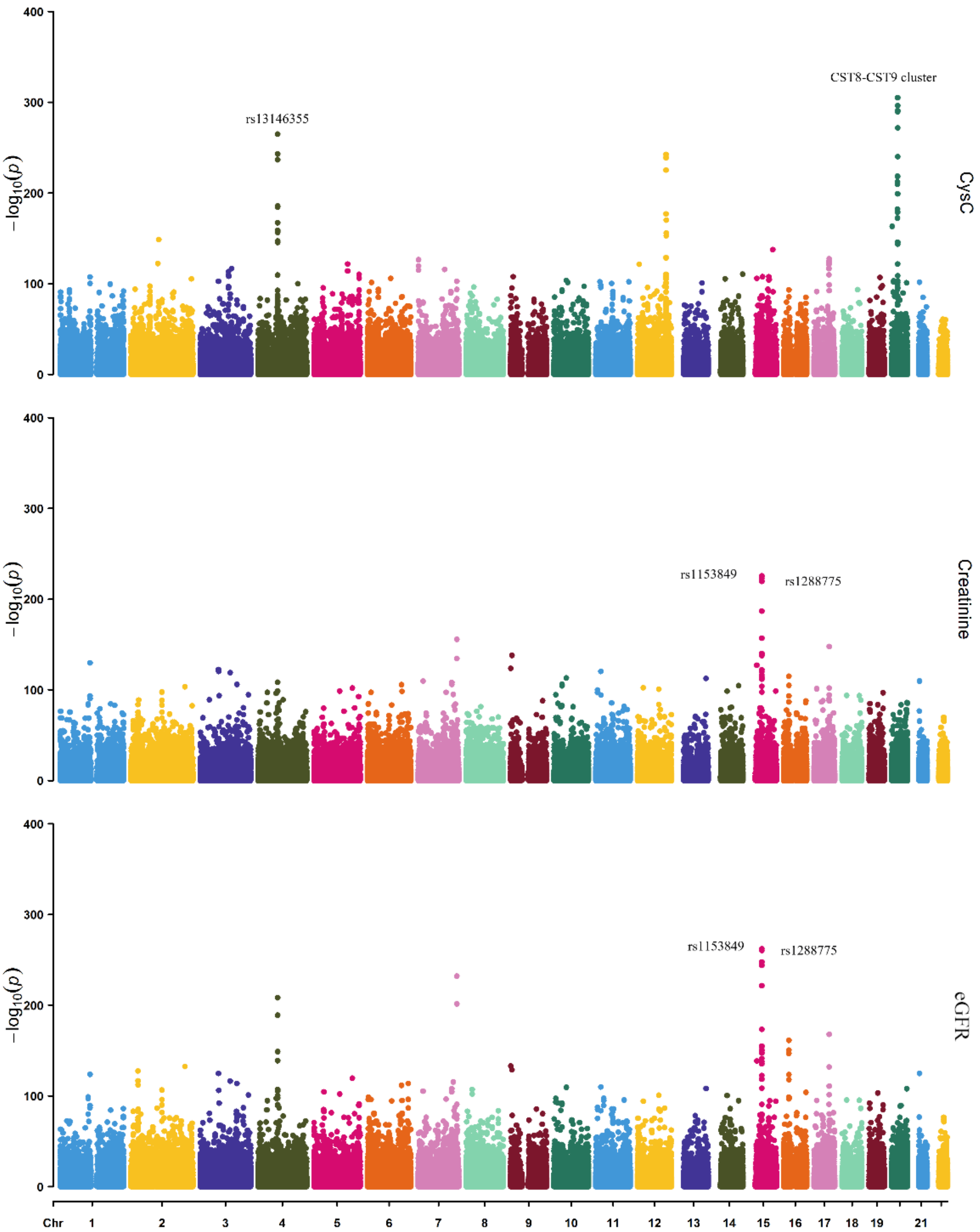

Figure S1. Manhattan map for genome-wide association analysis of CysC, SCr, and eGFR in UK Biobank subjects
